# Oral and Palmitate Paliperidone Long-Acting Injectable Formulations’ Use in Schizophrenia Spectrum Disorders: A Retrospective Cohort Study from the First Episode Psychosis Intervention Program (CRUPEP)

**DOI:** 10.1093/ijnp/pyab021

**Published:** 2021-05-02

**Authors:** R Segarra, M Recio-Barbero, M Sáenz-Herrero, O Mentxaka, J Cabezas-Garduño, J I Eguíluz, L F Callado

**Affiliations:** 1 Biocruces Bizkaia Health Research Institute, Cruces University Hospital, Bilbao, Spain; 2 Department of Psychiatry, Cruces University Hospital, Bilbao, Spain; 3 Department of Neurosciences, Faculty of Medicine and Nursing, University of the Basque Country (UPV/EHU), Leioa, Spain; 4 Department of Pharmacology, University of the Basque Country (UPV/EHU), Leioa, Spain; 5 Centro de Investigación Biomédica en Red de Salud Mental, CIBERSAM, Spain

**Keywords:** First episode psychosis, long-acting injectable antipsychotics, paliperidone, recent-onset psychosis, risperidone, schizophrenia spectrum disorders

## Abstract

**Background:**

Long-acting injectable antipsychotics (LAIs) may be a suitable therapeutic option for those patients in earlier stages of psychosis to avoid relapses and disease progression. Despite that, there is a lack of evidence in the literature regarding the use of LAIs in this profile of patients.

**Methods:**

This is a retrospective cohort analysis to assess the efficacy, tolerability, and pattern of use of palmitate paliperidone long-acting injectable (PPLAI) formulations (1- and 3-month doses) compared to oral paliperidone/risperidone in patients with a nonaffective first episode of psychosis (FEP) over 12 months of follow-up. Relevant sociodemographic and clinical information were assessed, as well as main clinical scales: Positive and Negative Syndrome Scale, Personal and Social Performance Scale, and Clinical Global Impression Scale Improvement and Severity measures.

**Results:**

The study included 48 patients, 16 per arm, who were aged 20–50 years and had an FEP. Significant improvements were registered for all treatment groups. Despite that, patients receiving PPLAI 1- and 3-month formulations obtained greater improvements than those in the oral group in the main domains assessed (*P* < .001). We found no statistically significant differences in hospitalizations between groups. Side effects were presented in 24% of patients. A trend towards reducing antipsychotic doses was observed in 43.8% of patients to achieve the minimum effective dose and avoid the occurrence of side effects.

**Conclusions:**

To our knowledge, this is the first study assessing the use of palmitate paliperidone long-acting formulations versus oral risperidone or paliperidone in FEP. Treatment with PPLAI formulations seems to be an effective therapeutic choice at earlier stages of the disease.

**Significance Statement**

This retrospective naturalistic cohort study examines the efficacy, tolerability, and patterns of use of oral and long-acting (1- and 3-month) paliperidone formulations over 12 months of follow-up in a sample of patients with a first episode of psychosis. To our knowledge, this is the first retrospective analysis explicitly comparing oral paliperidone with long-acting formulations in terms of effectiveness and functional ability in a sample of early-onset psychotic patients on antipsychotic monotherapy. Our results revealed a substantial improvement over time, with especially noteworthy improvement in the clinical and functional measures observed at the final assessment in patients receiving 3-month doses of long-acting antipsychotic treatment. Those results are of particular interest as functional recovery remains a cornerstone in patients’ long-term prognosis with psychosis. Likewise, in the context of an intervention program for a first episode of psychosis, we present evidence of real-world data of patients supporting the use of lower doses of antipsychotics than those frequently reported in the literature.

## Introduction

Schizophrenia includes some severe, chronic, and enduring diseases characterized by the presence of relapses and remission periods (Robinson et al., 1999). It is among the leading causes of disability worldwide and presents a substantial economic burden, with an annual estimated cost ranging from US $94 million to US $102 billion (Chong et al., 2016). When considering the debut of the disease, early intervention becomes a crucial period to avoid subsequent relapses and, thus, the progression of the disease (Birchwood et al., 1998). Up to 50% of patients with a first episode of psychosis (FEP) have incomplete remission of clinical symptoms, which are directly associated with the presence of long-term deficits in cognitive abilities, social functioning, and quality of life (Huber et al., 2008). Lack of insight and poor medication compliance have been widely described as 2 of the most important predictors associated with relapse and hospital readmissions (Chen et al., 2005; Novick et al., 2009). Nonadherence to antipsychotic treatment has been established as 1 of the best predictors of relapse in patients with psychosis (Karson et al., 2016; Mayoral-van Son et al., 2016; Hui et al., 2018). Moreover, the risk of relapse following a psychotic debut is estimated at around 77% within the first year and up to 90% in the following 2 years (Zipursky et al., 2013).

Failure to achieve remission of symptoms has been associated with the chronicity of the disease and persistent negative symptoms and functional deficits, which are crucial to achieving psychosocial recovery (Schooler, 2006). Chronicity has also been associated with the existence of frequent relapses, a higher burden of principal careers, an impact on direct and indirect costs of the disease, and poorer long-term course outcomes, including those directly associated with the daily psychosocial functioning of patients with an FEP (Álvarez-Jiménez et al., 2012b; Mayoral-van Son et al., 2019). Hence, functional recovery remains the cornerstone in managing and treating patients with an FEP (Robinson et al., 2004). Accordingly, the development of new therapeutic interventions has been focused on assessing the compliance and the efficacy of these treatments in order to provide better clinical and functional outcomes.

Although the effectiveness of current antipsychotic drugs for managing positive symptoms in schizophrenia is well-known, acute psychotic exacerbations are common in this profile of patients (Robinson et al., 1999; Emsley et al., 2013). Moreover, patients’ responses to antipsychotic treatment seem to decrease from the FEP to progressive illness relapses (Jäger et al., 2007; Takeuchi et al., 2019), and are associated with the presence of residual symptoms, hindering the goal of achieving functional recovery (Karson et al., 2016; Zipursky et al., 2018).

Over recent years, new antipsychotic formulations, such as long-acting injectable atypical antipsychotics (LAI-APs), have emerged as promising therapeutic choices. LAI-APs represent a suitable option for those patients presenting with an FEP, as they not only have the comfort of at least a single month/trimester dose, but also have improved treatment adherence. For that reason, and despite clinical guidelines not recommending LAI-AP formulations as a first-line treatment in psychosis, this therapeutic approach may represent a suitable option for those patients at earlier stages of the disease presenting with a lack of treatment adherence. As a result, some authors have proposed the use of LAI-AP formulations as the first line of treatment in the early stages of the disease, to avoiding the risks of relapse and resistance to drug treatments (Heres et al., 2014; Abdel-Baki et al., 2019; Salgueiro and Segarra, 2019).

To this day, there is scarce evidence in the literature regarding the use of LAI-APs as a first-choice line of treatment in patients with an FEP. The aim of this study was to evaluate the efficacy, tolerability, and patterns of use of long-acting injectable paliperidone palmitate formulations (PPLAI) compared to oral antipsychotic treatment in patients with an FEP.

## Methods

We conducted a retrospective, observational, naturalistic, 12-month study to assess the efficacy and tolerability of PPLAI formulations (1- and 3-month doses) compared to analogue oral antipsychotic formulations in a cohort of patients with nonaffective FEP. We defined oral antipsychotic treatment as the prescription of oral risperidone or paliperidone for comparison with PPLAI formulations, due to their similar pharmacological profiles.

The study population consisted of patients with an FEP who were taking oral risperidone or paliperidone or PPLAI formulations between May 2014 and December 2018. Patients from both groups were followed up through the First Episode Psychosis Intervention Program (CRUPEP Program) developed in 2004 at the Cruces University Hospital (Bilbao, Spain). The programmed frequency of outpatient visits was based on clinical conditions according to the protocolized CRUPEP Program, with minimum monthly assistance. Rehospitalization and emergency room attendance within 1 year of an antipsychotic medical prescription was also reviewed. Rehospitalization was indicated if the patient presented with a relapse of clinical symptoms or deteriorated functioning (Lehman et al., 2004).

Retrospective data were retrieved from the integrated electronic medical record system from the Basque Country Health Service. Relevant clinical and sociodemographic data and the number of resources used during the 12 months of follow-up (number of consultations carried out in the CRUPEP Program, hospitalizations, and emergency room attendances related to illness) were retrieved. Antipsychotic treatment was prescribed according to CRUPEP Program rationale, tailoring it to the specific necessities of each patient. We retrieved data from clinical scales evaluated as protocol, including the Positive and Negative Syndrome Scale (PANSS; Kay et al., 1987), the Personal and Social Performance Scale (PSP; Morosini et al., 2000), and the Clinical Global Impression Improvement and Severity scales (CGI-I and CGI-S; Busner and Targum, 2007).

The inclusion criteria for the study were being older than 18 years old and having a documented diagnosis of nonaffective FEP. The exclusion criteria for this study included having irregular control visits, having documented nonadherence to antipsychotic treatment, and withdrawal from follow-up consultations. Ethics committee approval was obtained for the development of this study. This research was performed in accordance with the ethical standards laid down at the Declaration of Helsinki. Conforming to international standards for research ethics, this study was approved by the local institutional ethical review committee.

### Statistical Analysis

Demographic and clinical baseline characteristics were summarized using descriptive statistics and were analyzed using a *t*-test for nominal variables or a Mann-Whitney U test. For categorical variables, the X^2^ test was used. Friedman’s test was used to test the significance of the change from baseline to the endpoint. Changes in clinical scales such as the PANSS and PSP were analyzed using the Wilcoxon signed-rank test and a paired *t*-test, respectively. Changes in the ordinal measures of CGI-S and CGI-I were analyzed using the Wilcoxon signed-rank test and a paired *t*-test. The level of statistical significance was set at *P* < .05. Data were analyzed using the IBM SPSS Statistical software, version 21.

## Results

### Sociodemographic Characteristics

**Figure 1.**
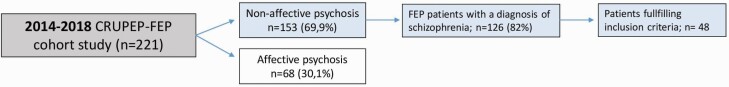
Description of the sample selection from the CRUPEP first-episode psychosis cohort. Abbreviation: CRUPEP, First Episode Psychosis Intervention Program; FEP, first episode of psychosis.

A total of 221 FEP patient records (both affective and nonaffective) were analyzed from the study time frame (2014–2018). Of them, 48 patients fulfilled the inclusion criteria of 12 months of treatment with oral risperidone or paliperidone monotherapy or with a 1-month or 3-month PPLAI monotherapy formulation, and patients’ clinical histories were retrieved for analysis. The flowchart for sample selection is presented in Figure 1.

Of the 48 included patients, 16 received oral risperidone or paliperidone, 16 received a 1-month dose of injectable paliperidone palmitate (PPLAI-1M), and 16 received a 3-month dose of injectable paliperidone palmitate (PPLAI-3M). Among them, 65% of all included patients were antipsychotic naïve until they started on paliperidone. For the patients who were not antipsychotic naïve, the median time from the debut of the disease to initiation of antipsychotic treatment with oral risperidone/paliperidone was 13 weeks (interquartile range, 2.65–19.5). Overall, patients did not present significant differences among the main sociodemographic variables assessed at baseline. Whereas the oral treatment group showed a higher proportion of women (50%), the difference did not reach statistical significance. In Table 1, we present the demographic baseline characteristics of all participants included in the study. Among other relevant sociodemographic variables, a substantial percentage of patients lived with their family of origin (58%) and were unemployed (62.5%), with these characteristics most pronounced in the PPLAI-1M and PPLAI-3M groups. At the end of the study, most patients had a diagnosis of schizophrenia (85%). Drug use was reported in 58% of patients, with cannabis (46%) being the most prevalent substance used among patients in the PPLAI-1M group, followed by stimulants (25%).

**Table 1.** Sociodemographic and clinical characteristics of patients receiving palmitate paliperidone long-acting injectable formulations or oral risperidone or paliperidone antipsychotics

	Oral risperidone or paliperidone (n = 16)	Paliperidone palmitate 1M (n = 16)	Paliperidone palmitate 3M (n = 16)	*P* value
Age, in years, mean (SD)	39.8 (9.89)	29.6 (9.67)	35.8 (6.67)	<.05
DUP, in weeks, mean (SD)	16.4 (13.9)	17.5 (20.1)	15.8 (12.3)	.991
Sex – male, n (%)	8 (50)	13 (81)	13 (81)	.080
Marital status – single, n (%)	11 (69)	10 (62.5)	10 (62.5)	.775
Living environment - family of origin, n (%)	9 (56)	12 (75)	7 (44)	.189
Laboral status – unemployed, n (%)	8 (50)	11 (69)	11 (69)	.449
Education level – secondary education, n (%)	11 (69)	14 (78)	12 (75)	.376
Main psychiatric diagnose – schizophrenia, n (%)	12 (75)	14 (87.5)	15 (94)	.310
Drug abuse – yes, n (%)	7 (44)	12 (75)	9 (56)	.196
Family psychiatric history, n (%)	6 (37.5)	7 (43.8)	5 (31.3)	.766
Antipsychotic monotherapy, n (%)	16 (100)	16 (100)	16 (100)	
Concomitant medication – benzodiazepines, n (%)	13 (81)	15 (94)	5 (31)	<.001

Abbreviations: DUP, duration of untreated psychosis; 1M, 1-month dose; 3M, 3-month dose; SD, standard deviation.

### Clinical Response

During the 12-month study period, the main domains assessed through the PANSS scale decreased in both the PPLAI-1M and oral groups. More specifically, the PPLAI-1M patients showed a significant reduction in scores on the PANSS positive subscale compared to those receiving oral risperidone or paliperidone (z = -4.708; *P* < .001). The mean changes from baseline to the final endpoint on the PANSS positive subscale were -10.81 (standard deviation [SD], 3.31) for the PPLAI-1M group and -6.56 (SD, 5.82) for the oral treatment group. As observed in Table 2, patients in the PPLAI-1M group showed better improvement in reducing the PANSS total score than those in the oral treatment group (*P* < .001). This improvement was also observed in the PPLAI-3M group, in which general maintenance of psychopathological stability was observed over time. It is worth noting that prior to starting PPLAI-3M treatment, patients must be clinically stabilized for at least 4 months at the same dosage of PPLAI-1M.

**Table 2.** Change in clinical and functional scales from baseline to endpoint in the oral and paliperidone palmitate long-acting injectable 1-month and 3-month treatment groups

	Oral risperidone or paliperidone, n = 16	Paliperidone palmitate 1M, n = 16	Paliperidone palmitate 3M, n = 16	*P* value
PANSS positive subscale, mean (SD)				
Baseline	14.69 (4.70)	18.19 (3.76)	7.56 (1.55)	.006
Change from baseline	-6.56 (5.82)	-10.81 (3.31)	-0.38 (1.26)	
PANSS negative subscale, mean (SD)				
Baseline	7.63 (1.41)	8.94 (2.02)	8.44 (1.83)	NS
Change from baseline	1.87 (3.90)	0.00 (3.05)	0.19 (2.26)	
PANSS general subscale, mean (SD)				
Baseline	23.88 (4.03)	25.00 (2.76)	17.63 (2.71)	<.001
Change from baseline	-5.56 (4.02)	-5.69 (3.36)	0.25 (1.95)	
PANSS total score, mean (SD)				
Baseline	46.19 (4.87)	52.44 (4.87)	34.63 (5.38)	<.001
Change from baseline	-10.25 (8.60)	-16.81 (5.56)	-1.06 (4.14)	
CGI-S, mean (SD)				
Baseline	4.06 (0.77)	5.13 (0.89)	2.50 (0.632)	<.001
Change from baseline	-1.37 (1.54)	-2.37 (1.09)	-0.06 (0.25)	
PSP score, mean (SD)				
Baseline	62.69 (10.47)	56.38 (7.65)	70.56 (6.90)	<.001
Change from baseline	7.19 (7.56)	16.62 (9.64)	7.56 (5.45)	

Abbreviations: 1M, 1-month dose; 3M, 3-month dose; CGI-S, Clinical Global Impression Severity; PANSS, Positive and Negative Syndrome Scale; PSP, Personal and Social Performance Scale; NS, not significant; SD, standard deviation.

#### CGI-I and CGI-S

During the 12-month study period, the severity of symptoms measured by the CGI-S scale significantly decreased in both the oral risperidone/paliperidone and PPLAI-1M groups (z = -4.63; *P* < .001). Similarly, scores on the CGI-I scale registered improvement both in the oral and PPLAI-1M patients (U = 48.50; *P* = .002).

**Figure 2.**
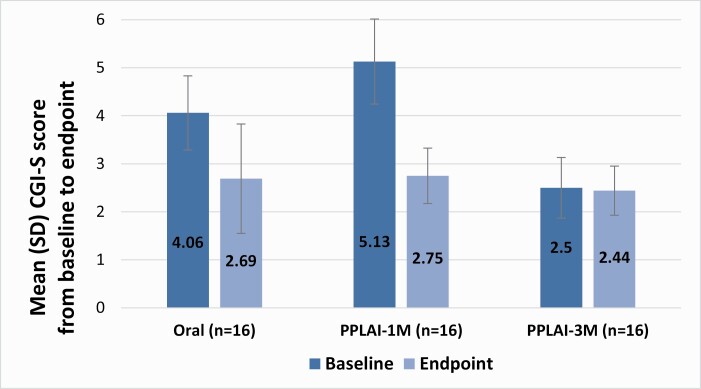
Changes between groups in CGI-S score from baseline to final assessment. Abbreviations: CGI-S, Clinical Global Impression–Severity of illness; Oral, oral risperidone or paliperidone antipsychotics; PPLAI-1M, paliperidone palmitate long-acting injectable 1-month dose; PPLAI-3M, paliperidone palmitate long-acting injectable 3-month dose; SD, standard deviation.

As shown in Table 2, patients in the PPLAI-1M group presented higher basal severity scores on the CGI-S scale compared to those in the oral group (*P* = .002). At the final assessment, patients in the PPLAI-1M group reached similar CGI-S scale results compared with patients taking oral antipsychotics. Despite that, the PPLAI-1M group obtained better improvement in the symptom severity scale, with a mean change from baseline of -2.37 (SD, 1.09) compared to -1.37 (SD, 1.54) in the oral arm. As observed in Figure 2, at the final assessment, patients in the PPLAI-3M group showed significant improvements in CGI-S and CGI-I scores compared to those in the oral group (z = 2.84; *P* = .004) and PPLAI-1M group (z = 3.55; *P* < .001).

#### PSP Scale

There were no statistical differences in PSP scores at baseline between groups. During the 12-month follow-up period, patients’ functioning measured through the PSP improved significantly from baseline to the endpoint in both the oral and PPLAI-1M treatment groups (t [31] = -6.89; *P* < .001). Despite that, as observed in Table 2, patients in the PPLAI-1M group showed significantly greater improvement in the PSP scale score, with a mean change from baseline to the endpoint of 16.62 (SD, 9.64).

**Figure 3.**
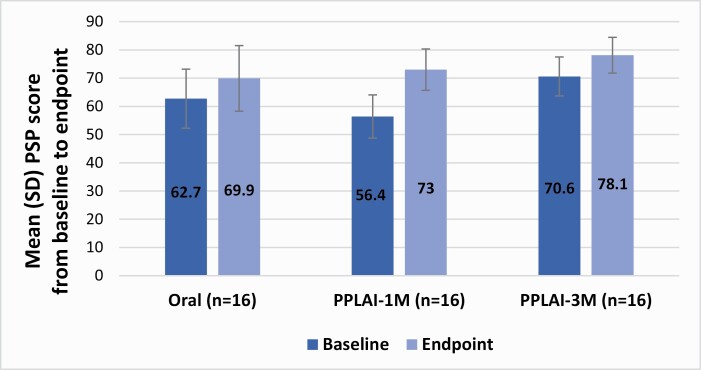
Mean changes between groups in PSP score from baseline to endpoint. Abbreviations: Oral, oral risperidone or paliperidone antipsychotics; PPLAI-1M, paliperidone palmitate long-acting injectable 1-month dose; PPLAI-3M, paliperidone palmitate long-acting injectable 3-month dose; PSP, Personal and Social Performance scale; SD, standard deviation.

Comparatively, patients receiving PPLAI-3M obtained greater PSP score results than those reported in the oral treatment group (t[31] = -2.24; *P* = .032). When compared with patients in the PPLAI-1M group, those in the PPLAI-3M had a statistically significant difference in PSP scores (t [31] = -4.689; *P* < .001) and a better level of functioning at the final assessment (Figure 3).

#### Health Resources Use

At baseline, 31 patients (64%) had a history of at least 1 psychiatric hospitalization before risperidone/paliperidone initiation. Comparatively, the PPLAI-1M group had a higher rate of the previous hospitalizations compared to other groups, which was statistically significant (44% oral; 94% PPLAI-1M; 56% PPLAI-3M; *P* = .009). During the 12-month follow-up, 3 (18%) patients in the oral group, 1 (6%) in the PPLAI-1M group, and 1 (6%) in the PPLAI-3M group were hospitalized. We did not find a statistically significant difference between groups (*P* = .409). The main reason for hospitalization was the inadequate management of clinical symptoms. None of the hospitalizations were due to severe adverse drug reactions. Concerning emergency room attendance, 3 patients in the oral group, 2 patients in the PPLAI-1M group, and 1 patient in the PPLAI-3M group required at least 1 visit to the emergency room during the 1-year study follow-up (*P* = .565).

#### Dosage

As shown in Table 1, all patients were on antipsychotic monotherapy. As a part of our FEP-CRUPEP Program, most patients were in treatment with low doses of oral antipsychotics, with baseline mean doses of 4.33 mg daily for oral paliperidone (n = 10; SD, 2.18 mg/day dosage range, 3–9 mg/day) and 3.50 mg daily for oral risperidone (n = 6; SD, 1.38 mg/day; dosage range, 2–4 mg/day). It should be mentioned that only 1 of the patients was on treatment with oral paliperidone at 9 mg/day due to clinical criteria. At the baseline assessment, the doses in patients from the PPLAI-1M group ranged from 75 mg to 150 mg, with a mean dose of 123.44 mg (SD, 28.09 mg). Finally, patients in the PPLAI-3M group were prescribed a mean dose of 328.19 mg (SD, 121.63) at baseline, with doses ranging from 175 mg to 525 mg. The main initiation doses of all patients receiving PPLAI after switching from oral treatment (n = 32) are presented in Table 3.

**Table 3.** Initiation dose strategy of all included patients on paliperidone palmitate depot formulations (n = 32) switching from oral risperidone or paliperidone to a 1-month dose of injectable paliperidone palmitate

Initiation dose			
Day 1	Day 8	Maintenance dose	n (%)
150 mg	100 mg	150 mg	10 (31.3)
100 mg	100 mg	100 mg	20 (62.5)
100 mg	75 mg	75 mg	1 (3.1)
75 mg	50 mg	50 mg	1 (3.1)

**Figure 4.**
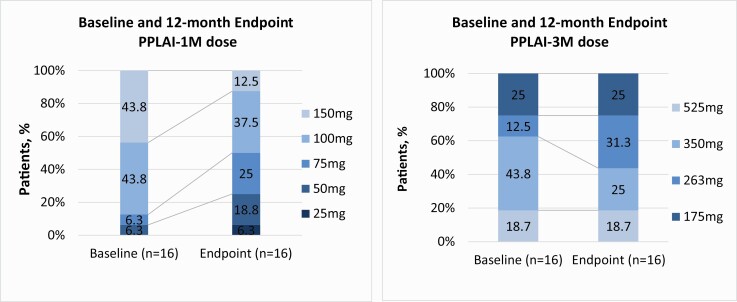
Prescribed paliperidone palmitate long-acting injectable 1-month and 3-month doses at baseline and the 12-month final assessment. Abbreviations: PPLAI-1M, paliperidone palmitate long-acting injectable 1-month dose; PPLAI-3M, paliperidone palmitate long-acting injectable 3-month dose.

At the final assessment, 21 patients (43.8%) reported a reduction of their initial antipsychotic dose and only 3 patients (6%)—2 in the oral group and 1 in the PPLAI-1M group—experienced a dose increase. At the endpoint, patients in the oral group received mean oral doses of 4.68 mg paliperidone (n = 10; SD, 2.18 mg/day; dosage range, 3–9 mg/day) and 2 mg risperidone (n = 6; SD, 0.75 mg/day; dosage range, 1–3 mg/day). Additionally, a reduction in the median dose was observed in the PPLAI-1M group (mean dose, 85.94 mg; SD, 34.12 mg; dosage range, 25–150 mg). Conversely, although a reduction of the antipsychotic dose was observed in the LAIPP-3M group, patients received a mean dose of 311.88 mg (SD, 123.55 mg; dosage range, 175–350 mg). Finally, patients in the PPLAI-1M group had a significant progressive reduction of the dose (z = -2.516; *P* = .112). Overall, a reduction in the standard doses of antipsychotics for all groups was observed across the study period (Figure 4). The low concomitant use of benzodiazepines in all groups, and mainly in the PPLAI-3M group, is also notable, as shown in Table 1.

#### Side effects

Treatment side effects were reported in 12 patients (24%), with the most described side effects related to sedation (42%), sexual dysfunction (40%), and weight gain (25%). In general, there were no statistical differences between groups, despite the PPLAI-1M group more frequently reporting an increased percentage of side effects, with the most prevalent being weight gain (25% oral; 33.3% PPLAI-1M; 12.5% PPLAI-3M, *P* = .363).

## Discussion

To our knowledge, this is the first study assessing the use of palmitate paliperidone long-acting 1- or 3-month formulations versus oral risperidone or paliperidone in a sample of patients with FEP. The results of our retrospective study show that both oral paliperidone or risperidone and PPLAI formulations are effective not only in the treatment of clinical symptoms but also in maintaining those improvements throughout at least 12 months. Despite that, there are some relevant differences between the 3 pharmacological groups that must be considered.

First, all patients seemed to achieve significant remission of clinical symptoms assessed through the PANSS scale. Despite that, it is necessary to consider that due to inner characteristics of the PPLAI-1M sample, patients in this group presented a higher baseline PANSS positive score. These results should be understood considering our study’s real-world setting, where patients in the PPLAI groups are often those who present with major clinical severity and treatment nonadherence. Thus, they could benefit from long-acting formulations to prevent lack of treatment adherence and consequent relapses.

Likewise, patients in both the PPLAI-1M and PPLAI-3M groups compared to those the oral treatment arm achieved significant improvements in all scales evaluated. Therefore, and due to the lack of studies assessing the efficacy of such formulations in patients with an FEP in a real-world setting, we tried to test whether patients in the PPLAI-3M group maintained clinical stability throughout 12 months of follow-up.

As a result, patients in the PPLAI-3M group obtained even better scores for general clinical symptom severity and saw a substantial improvement in the main domains of personal and social functioning, assessed through the PSP scale. Our data are consistent with those of previous studies reported in the literature, where PPLAI-3M formulation is recommended after clinical stability on PPLAI-1M for a minimum of 4 months (European Medicines Agency, 2016; Taylor and Huang, 2017). These results may be of particular relevance beyond the remission of acute symptoms, as functional recovery remains 1 of the main therapeutic goals to be achieved and involves patients’ overall day-to-day functioning, with particular importance for their long-term global prognosis after FEP onset (Álvarez-Jiménez et al., 2012a; Santesteban-Echarri et al., 2017).

We did not find statistically significant differences for either admissions or attendances at psychiatric emergency departments among groups. Despite that, out of the total number of people requiring psychiatric admission, 60% were in the oral treatment group. Moreover, considering that all patients included in the CRUPEP Program are monitored from the debut of their disease, this close protocolized follow-up could influence and contribute to lower rates of additional health resources use. As mentioned previously, the main reason for hospitalization was inadequate management of clinical symptoms. Thus, the prescribed doses of antipsychotics were adjusted according to clinical criteria.

It is noteworthy that more than 60% of the sample were antipsychotic-naïve patients, and all of them were on antipsychotic monotherapy at lower doses than those usually reported in the literature. This is our current clinical practice, according to the pharmacotherapeutic rationale intervention philosophy adopted in our FEP-CRUPEP Program.

As stated above, a trend towards reducing the antipsychotic dose is observed, to use the minimum effective dose to avoid the occurrence of unwanted side effects (Dixon and Stroup, 2015; Crespo-Facorro et al., 2016). Benzodiazepine administration is also exceptional and, if prescribed, benzodiazepines are sustained during a brief lapse of time according to a descendant dosage strategy (with a maximum of 12 weeks). A significant reduction of benzodiazepine use was observed in the LAIPP-3M group due to psychopathological stability and patients’ clinical improvements. We assume that the main reason for this result is derived from better and sustained clinical and functional outcomes, mainly in the PPLAI-3M group, which was able to reduce the anxiety levels derived from abnormal, psychotic experiences and difficulties in managing daily life events.

Despite the lack of literature regarding the effectiveness of long-acting antipsychotic formulations in patients with an FEP, our results are in line with those reporting the effectiveness of PPLAI-1M and PPLAI-3M formulations on the treatment of patients with early-onset psychosis (Emsley et al., 2008, 2017; Zhang et al., 2015; Bossie et al., 2017; Petrić et al., 2019).

Another handicap present in literature concerns the vast majority of published studies only considering patients with chronic schizophrenia. Only a few of them analyze the effectiveness of new antipsychotic formulations, including LAI formulations, in recent-onset psychosis and FEPs. Indeed, our research group has recently published a systematic review of the use of second-generation long-acting antipsychotics (LAI-SGA) in patients with an FEP. Among the main results, we conclude that treatment with LAI-SGAs could offer several advantages over oral treatment in FEPs, such as treatment adherence and relapse prevention due to treatment discontinuation (Salgueiro and Segarra, 2019).

Accordingly, we present here the first study assessing the use of PPLAI formulations, in 1-month or 3-month doses, versus oral risperidone or paliperidone in an FEP sample, trying to confirm our former hypothesis empirically.

As previously mentioned, due to the particularities of daily clinical practice, patients in the PPLAI-1M group frequently had more severe symptoms, presenting lack of insight, treatment nonadherence, and substance use. Treatment noncompliance remains a key issue in patients with schizophrenia. Thus, long-acting formulations seem to present a therapeutic option for those patients that present with an increased risk of treatment discontinuation and relapse (Kane and Garcia-Ribera, 2009; Arango et al., 2019). Moreover, including PPLAI formulations seems to be an effective therapeutic choice for treating clinical symptoms and improving the psychosocial burden of the disease, even in the early stages an FEP, in patients that voluntarily accept and consider such a therapeutical option after giving adequate informed consent. LAI treatment has emerged as an alternative to oral formulations to improve treatment adherence and reduce the risk of relapse and, thus, chronic states of the disease (Fagiolini et al., 2017). Despite that, and considering the potential therapeutic benefits of these formulations, the rate of LAI prescribing remains low (Arango et al., 2019).

Our study has several limitations. First of all, the sample size is small, which limits the generalization of the results. The study is a retrospective, observational study designed to explore treatment outcomes and is limited by criteria selection. Due to the inclusion criteria, patients were required to have been on treatment with an oral antipsychotic (risperidone or paliperidone) or with a PPLAI 1-month or 3-month formulation for at least 12 months; therefore, there could be bias among groups, as patients using LAI formulations are adherent to antipsychotic treatment, while real adherence to oral antipsychotic treatment is unknown. In this regard, our research group recently published a study testing plasmatic blood concentrations of the main oral second-generation antipsychotics in an FEP sample, pointing out that nearly half of the enrolled patients were nonadherent to oral antipsychotic treatment or presented subtherapeutic levels at 1 year of follow-up (Bustillo et al., 2018). Additionally, as discussed above, the close clinical monitoring of all patients included in our FEP program may have resulted in lower hospitalization rates and less clinical resource use. Considering these limitations, the outcomes of this study are in need of further confirmation due to the lack of studies addressing the use of LAI formulations in patients with an FEP.
